# Clathrin mediated endocytosis is involved in the uptake of exogenous double-stranded RNA in the white mold phytopathogen *Sclerotinia sclerotiorum*

**DOI:** 10.1038/s41598-020-69771-9

**Published:** 2020-07-29

**Authors:** Nick Wytinck, Daniel S. Sullivan, Kirsten T. Biggar, Leandro Crisostomo, Peter Pelka, Mark F. Belmonte, Steve Whyard

**Affiliations:** 1 Department of Biological Sciences, University of Manitoba, Winnipeg, R3T 2N2 Canada; 2 Department of Microbiology, University of Manitoba, Winnipeg, R3T 2N2 Canada

**Keywords:** Endocytosis, RNAi, Plant sciences

## Abstract

RNA interference (RNAi) technologies have recently been developed to control a growing number of agronomically significant fungal phytopathogens, including the white mold pathogen, *Sclerotinia sclerotiorum*. Exposure of this fungus to exogenous double-stranded RNA (dsRNA) results in potent RNAi-mediated knockdown of target genes’ transcripts, but it is unclear how the dsRNA can enter the fungal cells. In nematodes, specialized dsRNA transport proteins such as SID-1 facilitate dsRNA uptake, but for many other eukaryotes in which the dsRNA uptake mechanisms have been examined, endocytosis appears to mediate the uptake process. In this study, using live cell imaging, transgenic fungal cultures and endocytic inhibitors, we determined that the uptake mechanism in *S. sclerotiorum* occurs through clathrin-mediated endocytosis. RNAi-mediated knockdown of several clathrin-mediated endocytic genes’ transcripts confirmed the involvement of this cellular uptake process in facilitating RNAi in this fungus. Understanding the mode of dsRNA entry into the fungus will prove useful in designing and optimizing future dsRNA-based control methods and in anticipating possible mechanisms by which phytopathogens may develop resistance to this novel category of fungicides.

## Introduction

Fungal phytopathogens cause billions of dollars of crop losses each year, despite our efforts to control them using a spectrum of chemical fungicides^1^. With continued use of these chemicals, there are increasing concerns of their negative impacts on off-target species. The fungicide vinclozolin, for example, was recently banned due to concerns of threats to human health^2^, while strobilurin fungicides have been observed to adversely affect non-targeted soil microorganisms and aquatic animals if runoff occurs after spraying^3^. The frequent use of various fungicides has also resulted in the development of resistant fungi for multiple classes of these chemicals^4–6^. New, more species-specific fungal control strategies are clearly needed. RNA interference (RNAi)-based technologies are emerging as viable and promising alternative control strategies for a growing number of fungal phytopathogens. Due to RNAi’s sequence specificity, double-stranded RNA-based fungicides could provide species-limited control of pathogenic fungi without adversely affecting non-target organisms^7^.

RNAi-based fungal pathogen protection in plants can be achieved through a transgenic approach, where the host plant produces hairpin (hp)RNAs that can induce transcript knockdown in an invading pathogen. This so-called host-induced gene silencing (HIGS) has been observed to provide effective protection in tobacco^8,9^, cotton^10^, banana^11^, barley and wheat^12,13^. While HIGS can provide a consistent level of resistance for these plants, the requirement for the development of transformation protocols for each plant species, as well as the public’s perception of genetically modified organisms may limit its application in many commercial crops^14^.

A more versatile dsRNA delivery system that could be applied to any plant is spray-induced gene silencing (SIGS), where dsRNAs are applied topically. The application of SIGS was first demonstrated as a viable method of fungal control for the cereal pathogen *Fusarium graminearum*^15^. In that study, SIGS was used to deliver to barley plants a dsRNA molecule that simultaneously targeted three *Fusarium* cytochrome P450 lanosterol C-14-α demethylase genes' tanscripts. This dsRNA was effective at knocking down the three mRNA targets and significantly reduced pathogen growth on the host plant. Interestingly, the topically-applied dsRNA appeared to spread over and through the barley leaves; the molecules were detected within the plant’s vasculature and as such, *Fusarium*’s conidial germination was also inhibited on unsprayed distal portions of the leaves. SIGS was also found to be effective in the control of *Botrytis cinerea,* a necrotrophic fungus that is particularly destructive to horticultural crops. Wang et al.^16^ designed dsRNA molecules to target the Dicer-like (DCL) 1/2 transcripts in this fungus, which reduced production of small RNAs that inhibit host plant defense genes^17^. By interfering with the fungus’ ability to produce these pathogenicity factors, Wang et al. demonstrated reduced infection of a wide range of hosts including strawberries, grapes, lettuce and tomatoes.

With these initial studies demonstrating the potential of this technology to control fungal pathogens, increased efforts to identify novel gene targets in other phytopathogen species followed. McLoughlin et al.^18^ developed an RNAi target identification pipeline using the *Brassica napus–Sclerotinia sclerotiorum* pathosystem as a template. A suite of novel gene targets effective at limiting the pathogenicity of this aggressive necrotroph was established through the interrogation of transcriptomic data and functional bioassays, which screened putative dsRNA targets using mature leaf infection assays with Sclerotinia-inoculated petals. The applicability of this target identification system in other species was further demonstrated by effectively targeting homologous genes’ transcripts in *B. cinerea*. In each of these fungal species for which SIGS-induced RNAi has been observed, the dsRNA evidently entered the fungal cells to initiate the RNAi-mediated knockdown of target transcripts. How the dsRNA enters the fungal cells was unknown, but understanding the mechanism(s) could prove invaluable in future efforts to improve dsRNA uptake for enhanced RNAi efficacy, as well as to identify any possible barriers to dsRNA uptake for applications in a broader range of fungi^14^. For example, in different insect orders, there is a gradient in the efficiency of RNAi responses, with coleopterans (beetles) generally showing high susceptibility and lepidopterans (moths) being much more refractory to dsRNA. In the lepidopterans, reduced sensitivity was attributed to both nuclease-mediated degradation of dsRNA as well as entrapment of dsRNA within the cells’ endosomes, resulting in failure to enter the cytoplasm to reach the mRNA targets^19^. Failure of cells to take up dsRNA has also been observed in a lab-selected dsRNA-resistant strain of corn rootworm *Diabrotica virgifera,* although the mechanisms restricting uptake have not yet been identified^20^.

Uptake of dsRNA into cells has been more thoroughly studied in invertebrates. The nematode *Caenorhabditis elegans* was the first organism to have RNAi described, and in these worms, several dsRNA transmembrane proteins, known as systemic RNA interference defective (SID) proteins, have been observed to facilitate dsRNA uptake^21–25^. RNAi has since been applied widely in many species including various pest insects, where dsRNAs are being developed as novel biocidal molecules that target essential genes, like those in the pathogenic fungi described above. In most insect control applications, the dsRNAs are intended to be incorporated into or sprayed on their food source, and hence, uptake of the dsRNA will occur through the intestinal cells^26^. In some species of beetles, SID1-like (SIL) proteins have been identified, but their role in dsRNA uptake is unclear, as knockouts of these genes in some species had no impact in RNAi efficacy^27^. In the fruit fly *Drosophila melanogaster,* orthologs for SID1 or SIL proteins do not exist^28^. Instead, genetic screening of S2 cells in *Drosophila* identified clathrin-mediated endocytosis (CME) as a likely route for dsRNA uptake^29,30^. CME has since been found to be the primary mode of uptake in various other insect species, including *Tribolium castaneum*,* Bactrocera dorsalis* and *D. virgifera*^31–33^. In this process, dsRNA binds to an as yet unidentified receptor, induces the formation of a clathrin-coated pit that invaginates to form a vesicle around the dsRNA. The vesicle is then transported through the endosomal compartment, and eventually is released from the endosome via pH shifts generated by proton pumps. While insect species such as *Leptinotarsa decemlineata* use both SIL channels and CME to take up dsRNA, the relative contributions of each remain unclear^34^.

Despite these advances in insect systems, the dsRNA uptake mechanisms have not been resolved in fungal species. Understanding how exogenously-applied dsRNAs enter fungal cells is of critical importance in developing the technology for commercial applications. For example, understanding uptake mechanisms can help identify potential rate-limiting barriers to RNAi, either in dsRNA-insensitive strains or species, or in strains that might develop resistance due to changes in uptake mechanisms. Secondly, understanding uptake will help in identifying suitable formulations for SIGS, to maximize retention of dsRNA on the plant while still facilitating dRNA uptake by the fungus. In this study, we explored the role of dsRNA uptake via CME in the fungus *Sclerotinia sclerotiorum*. Using both chemical and RNAi-based methods of inhibiting endocytic processes, we evaluated the uptake of dsRNA into growing *S. sclerotiorum* hyphae. With the aid of live cell imaging and confocal microscopy, coupled with measurements of RNAi-mediated knockdown of reporter genes within the fungal cells, we show that proteins associated with CME are essential for dsRNA uptake. Taken together, our findings provide insight into the mode of action of exogenously-applied dsRNAs in fungal cells through SIGS, and how RNAi may provide effective control of agronomically important fungal pathogens.

## Results

### Visualizing regions of dsRNA uptake

**Figure 1:**
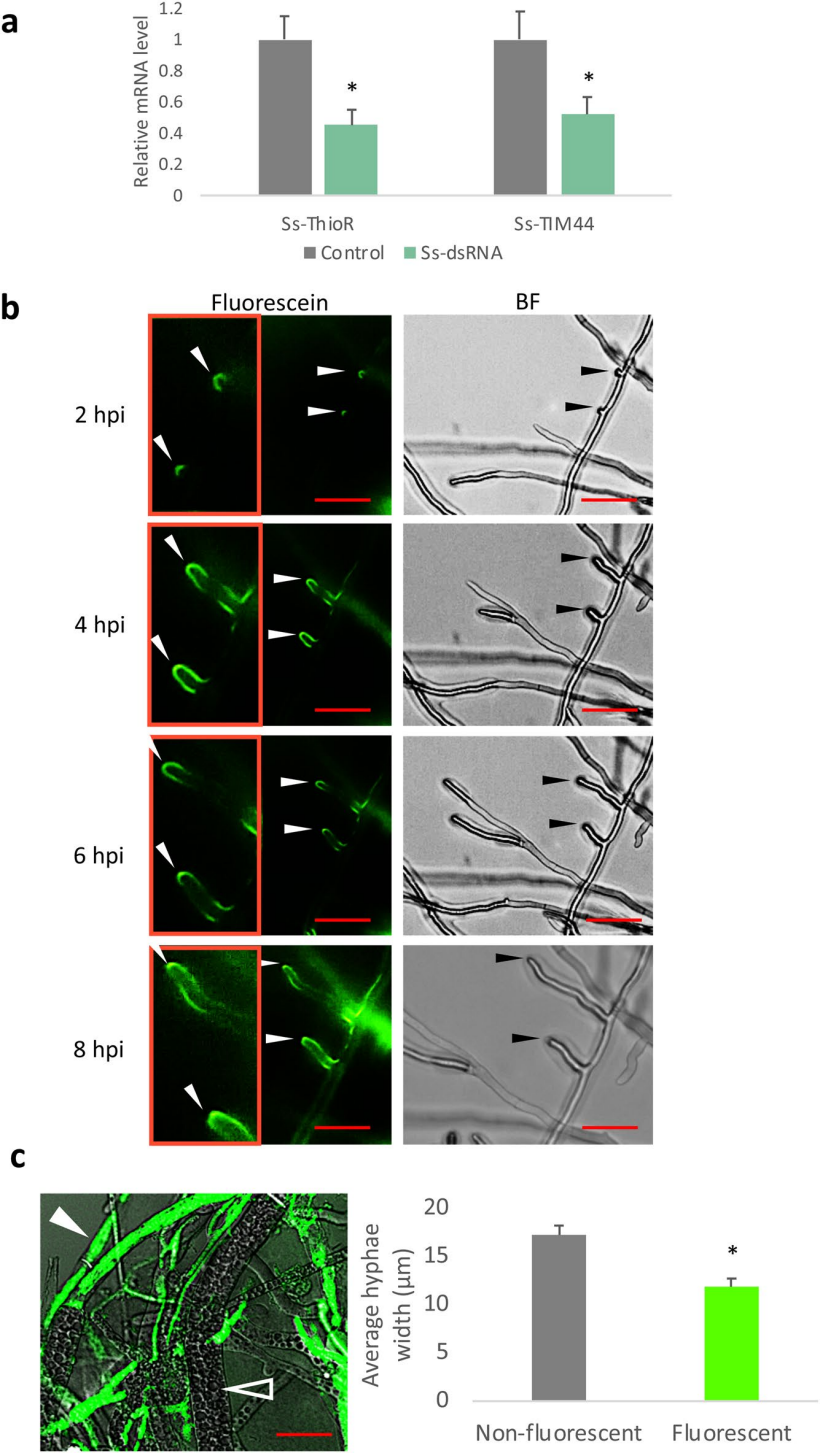
Uptake of dsRNA by Sclerotinia. (**a**) In vitro cultures were treated with 500 ng/mL of dsRNA targeting two Sclerotinia genes, Ss-ThioR and Ss-TIM44. Transcript abundance was measured after 48 h. Asterisk denotes significant reduction in transcripts, relative to the controls (one-tailed t-test, p < 0.05 from three independent biological replicates) (**b**) Detection of fluorescein-tagged Ss-ThioR dsRNA in newly germinated hyphae from ascospores. Growing hyphae were imaged using the ImageXpress platform. At each time point, representative bright-field (BF) images (right) reveal growing hyphae (arrow heads), and their fluorescence is visualized in the middle panel. Enlarged images of the hyphal tips are shown in the left panel (Scale bars: 40 µm). (**c**) Three day-old *Sclerotinia* inoculated with fluorescein-labelled dsRNA show different levels of fluorescence 8 h later. (Hollow arrow with white border: mature, vacuolated hyphae; white arrow: younger hyphae, more prone to uptake). The widths of all hyphae were measured at their midpoints, and were scored for absence or presence of fluorescence (Scale bars: 20 µm). Asterisk denotes significant difference in the width of fluorescent hyphae, relative to non-fluorescing hyphae (one-tailed t-test, p < 0.05; n = 40 hyphae of each category).

Liquid cultures of Sclerotinia hyphae, when incubated with dsRNAs targeting transcripts of either thioredoxin reductase (Ss-ThioR) or Ss-TIM44, a gene encoding a mitochondrial inner membrane transporter, showed reduction of either gene’s transcripts by approximately 50% after 48 h (Fig. 1a). Clearly, both dsRNAs could readily enter the fungal cells to induce RNAi and there does not seem to be significant differences related to extent of knockdown for the two different dsRNA targets.

To visualize the uptake of exogenous dsRNA in Sclerotinia, ascospores were germinated and then inoculated and allowed to grow in the presence of fluorescein-labeled Ss-Thio-R dsRNA. The distribution of labeled RNA was examined over an 8 h time period. The fluorescently-labeled dsRNA was detectable on or inside the fungal cells within two hours of application of the dsRNA (Fig. 1b). Interestingly, the fluorescence accumulated at specific sites, initially localized on the newly budding branches, and over the next several hours, the fluorescence was concentrated at the tips of these branches and at the junction points of the branches and the main trunk of the hyphae. Examination of the focal stacks of the images revealed fluorescence throughout the hyphal tips and branch points, suggesting that the fluorescently-labeled dsRNA was not on the surface, but was localizing within the cytoplasm of the cells at these locations (Supplementary Fig. S1).

While newly-germinated spores showed rather localized concentration of the fluorescently-labeled dsRNA, the distribution of the fluorescence in 3-day old hyphae that were exposed to dsRNA for 8 h showed more widespread distribution of the dsRNA. Confocal microscopy revealed that the fluorescence was not evenly dispersed throughout all hyphae, as thinner, younger hyphae had higher levels of fluorescence than the thicker, more mature and vacuolated counterparts (Fig. 1c).

### Chemical inhibition of endocytic processes

**Figure 2:**
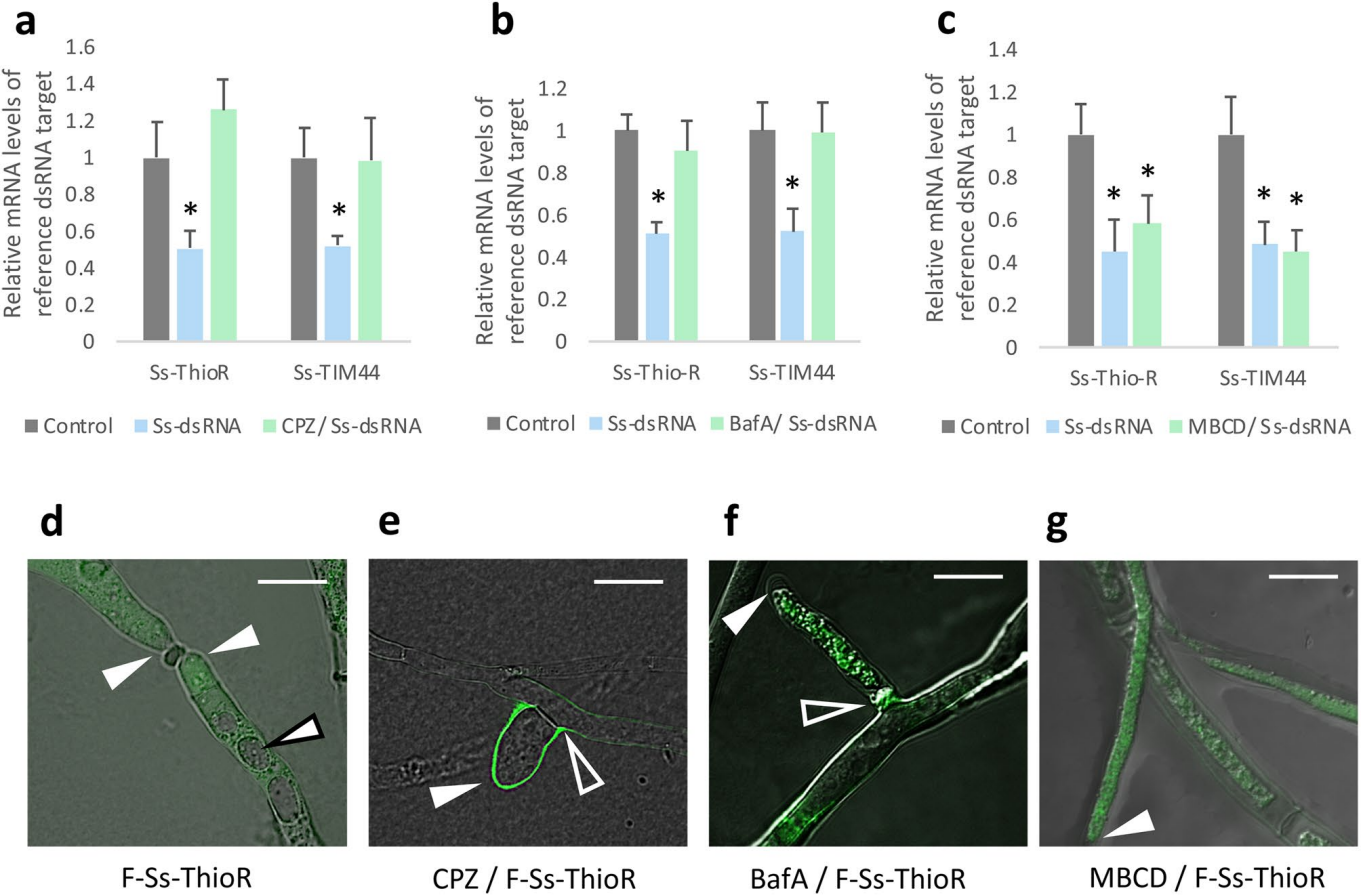
Chemical inhibition of clathrin mediated endocytosis inhibits dsRNA uptake. (**a**) In vitro cultures were inoculated with 20 µM chlorpromazine (CPZ), (**b**) 0.2 µM bafilomycin A1 (BafA), or (**c**) 2 mM methyl-beta-cyclodextrin (MBCD). Two hours later, hyphae were treated with 500 ng/mL Ss-ThioR or Ss-TIM44 dsRNA and transcript levels of these two genes were measured two days later. Asterisk denote significantly different values relative to the negative control (one-tailed t-test, p < 0.05, from three independent biological replicates). (**d**–**g**) Confocal microscopy reveals distribution of fluorescein-labelled dsRNA in hyphae, 4 h post-treatment, treated with or without inhibitors. (**d**) No inhibitors, (**e**) 20 µM CPZ, (**f**) 0.2 µM BafA, or (**g**) 2 mM MBCD. Cultures were imaged three separate times with similar results. (White arrow with black border: fungal vacuole; White arrow with no border: hyphal tip; Hollow arrow with white border: branch point of a hyphal bud). (Scale bars: 10 µm).

To examine the possible role of clathrin mediated endocytosis (CME) or clathrin-independent (e.g. caveolae-mediated endocytosis) in dsRNA uptake in Sclerotinia, hyphae were grown in liquid cultures dosed with different chemical inhibitors of endocytosis and then treated with dsRNAs targeting ThioR or TIM44. Hyphae treated with chlorpromazine (CPZ), which inhibits the recruitment of clathrin to the plasma membrane and thus inhibits the formation of vesicles, or bafilomycin A1 (BafA), which impedes the function of vacuolar ATPases and therefore prevents the release of vesicular cargo into the cytoplasm, failed to induce RNAi-mediated knockdown of either Ss-ThioR or Ss-TIM44 (Fig. 2a,b). This lack of transcript knockdown suggests that CME is playing a significant role in dsRNA uptake in the Sclerotinia hyphae. A third inhibitor, methyl-beta-cyclodextrin (MBCD), which inhibits clathrin-independent processes such as caveolae mediated endocytosis and lipid raft formation, was also tested. Interestingly, both target genes’ transcript levels were reduced when treated with their respective dsRNAs after this inhibitor treatment (Fig. 2c). This suggests that clathrin-independent uptake processes are not playing a major role in dsRNA uptake in the fungus.

Confocal microscopy was used to detect the fluorescently-labeled dsRNAs after treatment with the different inhibitors. Hyphae from liquid cultures treated only with fluorescently-labeled dsRNAs were readily able to internalize the molecules. The labeled dsRNA remained cytoplasmic, with no fluorescent signal appearing within the vacuole structures (Fig. 2d). CPZ treatment completely prevented the labeled dsRNAs from entering the cells, as all fluorescence was external, with the strongest signal appearing around the outside of newly budded branches (Fig. 2e). Bafilomycin-treated cells were able to internalize the fluorescence, however, as evidenced by the lack of transcript knockdown, the dsRNA was not released from the internal vesicles (Fig. 2f). It is interesting that there was no obvious cell-to-cell spread of the dsRNA over the 4 h experiment, and the fluorescent signal appears quite punctate. The distribution of the fluorescence in the MBCD-treated cells did not appear to differ greatly from that of the untreated cultures (Fig. 2g). These cell images therefore support the premise that CME is a major contributor to dsRNA uptake.

### RNAi of RNAi experiment to elucidate candidate uptake genes

**Figure 3:**
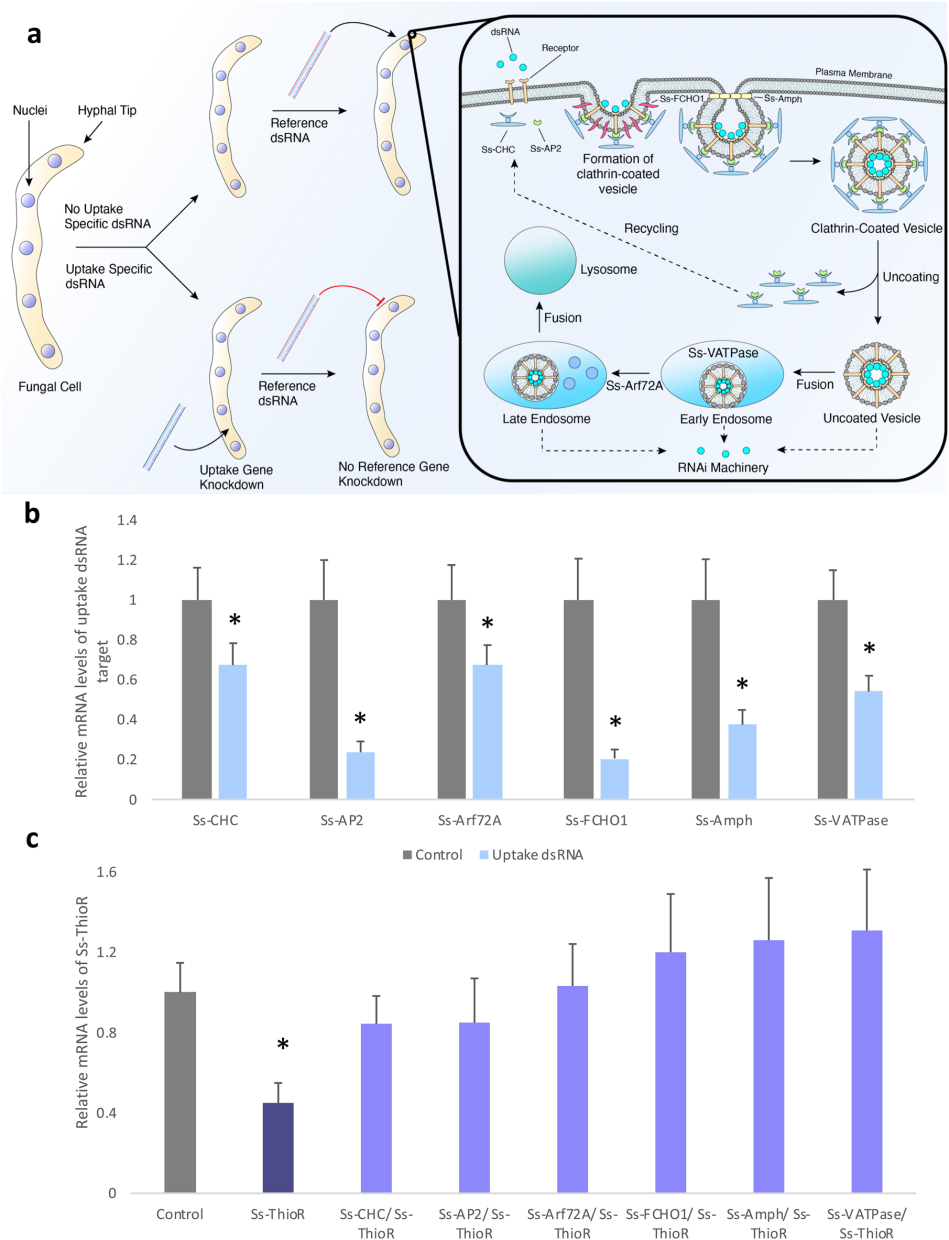
An RNAi of RNAi experiment to identify proteins involved in dsRNA uptake. (**a**) The knockdown of genes involved in uptake will inhibit further uptake of reference dsRNA and thus prevent its knockdown. Whereas in cultures untreated with uptake dsRNA, reference dsRNA will be taken up via our proposed uptake mechanism. The binding of exogenous dsRNA to its receptor induces the localization of clathrin and its adaptor to the membrane. FCHO contributes to the invagination of the vesicle and amphiphysin to its release from the membrane. Arf72A aids in the transition from early to late endosome, and vesicle acidification via VH + ATPase induces cytoplasmic dsRNA release. (**b**) In vitro cultures were first treated with 500 ng/mL of dsRNA targeting putative uptake genes (Ss-CHC, Ss-AP2, Ss-Arf72A, Ss-FCHO1, Ss-Amph and Ss-VATPase) and transcript levels of each gene was assessed by quantitative real-time PCR 4 days later. Asterisk denotes significant knockdown of the targeted gene (one-tailed t-test, with a Bonferroni correction p < 0.05 from three independent biological replicates). (**c**) At day 4, a second dsRNA, targeting Ss-ThioR, was applied, and Ss-ThioR transcript levels were measured after an additional two days. Asterisk denotes significant difference relative to the negative control (one-tailed t-test with Bonferroni correction, p < 0.05 from three independent biological replicates). Note that only the hyphae treated with Ss-ThioR dsRNA alone resulted in significant difference from the controls.

To validate that CME is facilitating dsRNA uptake, an ‘RNAi of RNAi’ experiment was conducted (Fig. 3a). In these experiments, cultured cells were first treated with a dsRNA targeting a gene putatively involved in dsRNA uptake. After sufficient time had passed to permit RNAi-mediated knockdown and reduction of the targeted gene’s protein, cultures were treated with a secondary, reference dsRNA. If the reference dsRNA did not knock down its target transcript to the same extent as untreated or negative controls, it is evidence that the second dsRNA failed to enter the cells, and thus confirms the involvement of the initial dsRNA target in uptake.

Six Sclerotinia genes with high sequence identity to genes encoding proteins annotated to function in clathrin mediated endocytosis in *Aspergillus clavatus* were identified and dsRNAs were prepared to target each*.* Sclerotinia liquid hyphae cultures pretreated for 4 days with each of these dsRNAs [clathrin heavy chain (Ss-CHC); vacuolar H^+^ ATPase 16 kDa subunit (Ss-VATPase); ADP ribolysation factor 72A (Ss-Arf72A); Fes/CIP4 homology domain only 1 (Ss-FCHO1); amphiphysin (Ss-Amph); and clathrin adaptor protein 2 subunit A2 (Ss-AP2)] showed significant knockdown of each of the six targets (Fig. 3b). Subsequent treatments with the reference dsRNA, Ss-ThioR, failed to induce gene knockdown (Fig. 3c). This provides strong evidence in favour of the involvement of these genes, and therefore CME in dsRNA uptake in Sclerotinia.

### Transgenic Sclerotinia encoding eGFP to validate CME as the primary mode of uptake

**Figure 4:**
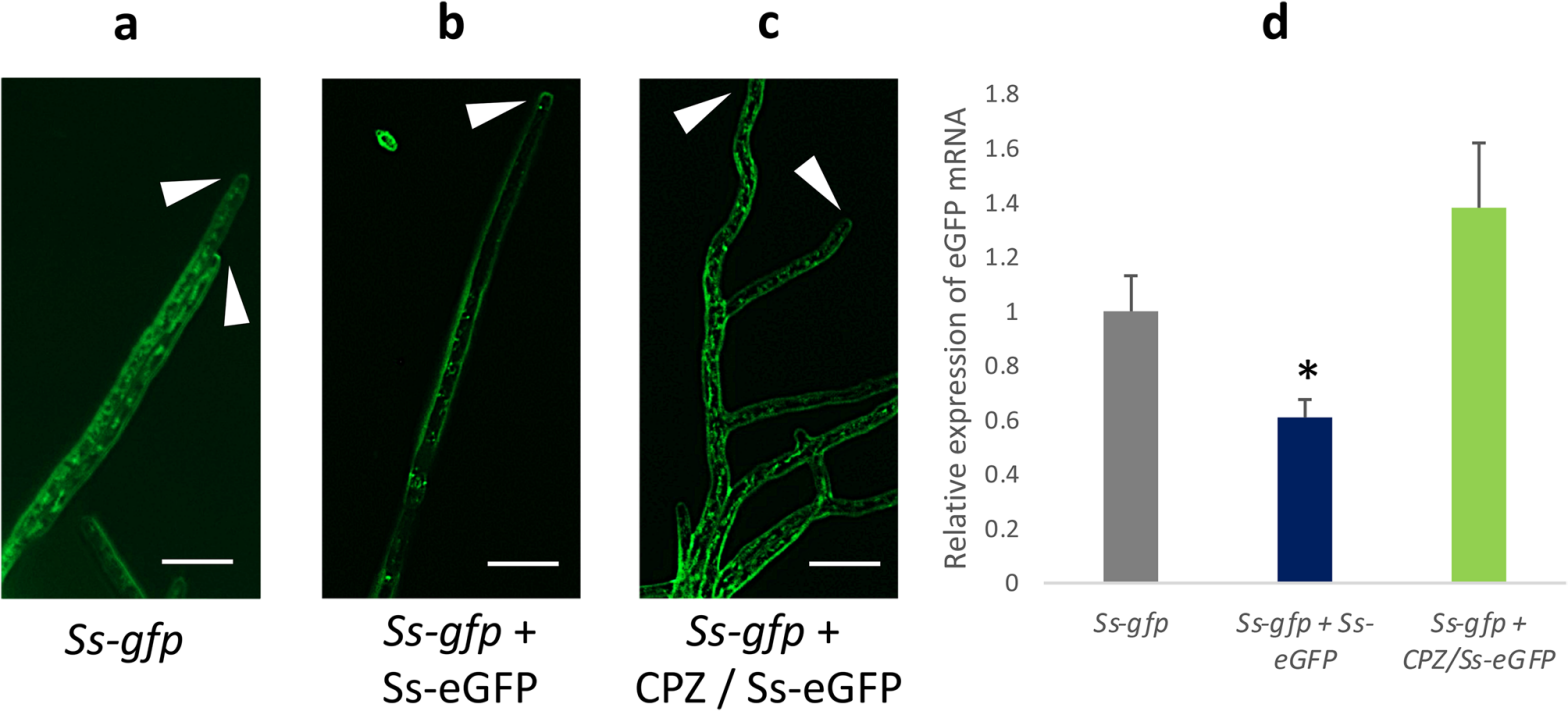
EGFP-transgenic Sclerotinia show reduced fluorescence when treated with dsRNA targeting eGFP, but not when first treated with chlorpromazine (CPZ). In vitro cultures of the eGFP-Sclerotinia were treated with 1,000 ng/mL of dsRNA targeting eGFP with or without 20 µM CPZ. After four days, hyphae were imaged. (**a**) Untreated eGFP-Sclerotinia showing normal fluorescence (White arrows with no border: hyphal tips) (**b**) eGFP Sclerotinia treated with eGFP dsRNA. Note stronger loss of fluorescence at hyphal tips. (**c**) CPZ-treated eGFP-Sclerotinia treated with eGFP dsRNA (Scale bars: 20 µm). (**d**) eGFP transcript levels in each of the treatment. Asterisk denotes significant difference from the negative control (one-tailed t-test, p < 0.05 from three independent biological replicates).

Further evidence that CME is a primary means of dsRNA uptake was demonstrated using transgenic Sclerotinia that constitutively expressed eGFP (Fig. 4a). Liquid cultures of the eGFP strain, when treated with dsRNA targeting the eGFP gene’s transcripts, had reduced fluorescence. Intriguingly, the region behind hyphal tip, where fluorescein-labeled dsRNA had previously been observed to localize, showed the strongest reduction in eGFP fluorescence, which suggests that this region could be the point of entry of dsRNA, or alternatively, is a site where the dsRNA preferentially localizes as it induces knock down (Fig. 4b). Cultures treated with eGFP dsRNA also showed significantly reduced eGFP mRNA levels (Fig. 4d). In order to demonstrate that the eGFP dsRNA entered via clathrin mediated endocytosis, the liquid cultures were treated with chlorpromazine. Hyphae treated with CPZ prior to eGFP dsRNA addition did not display a reduction in fluorescence, indicating that the dsRNA was unable to enter the hyphae (Fig. 4c). Transcript reductions of eGFP mRNA also were not observed in these CPZ-treated cultures (Fig. 4d).

## Discussion

RNAi-based fungicides offer great potential for managing some of our crops’ most damaging pathogens. For the technology to reach its fullest potential, we will need to have a good grasp of the molecular processes involved in dsRNA uptake in fungi, to achieve effective dsRNA delivery and potent RNAi. Our findings demonstrate how exogenously-applied dsRNAs can enter fungal cells to initiate RNAi-based gene knockdown, which we and others are considering for use in both gene validation studies and control of this pathogenic fungus in the field. In this study, we used chemical inhibitors, targeted gene knockdown, and live cell imaging to demonstrate that clathrin-mediated endocytosis (CME) plays a major role in the uptake of exogenously-applied dsRNAs in the white mold pathogen *S. sclerotiorum.*

Chemical inhibitors have been widely used to examine dsRNA uptake in a variety of insect species^29,31,34^, and in this study, two inhibitors of CME, chlorpromazine (CPZ) and bafilomycin A (BafA), confirmed a role for CME in dsRNA uptake through the reduction of the cell’s RNAi response to the two reference genes. CPZ is a cationic amphiphilic compound that prevents the formation of clathrin-coated pits at the site of vesicle invagination by translocation of clathrin from the plasma membrane to intracellular vesicles^35^. Visualization of the CPZ-treated hyphae using confocal microscopy indicated that the fluorescein-labeled dsRNA failed to enter the cells, but instead was concentrated on the cell’s exterior, possibly binding to the positively charged chitin in the cell wall^36^. The use of the fluorescein dye to label the dsRNA did not likely impair dsRNA uptake, as fluorescently-labeled dsRNAs have been demonstrated to enter other cell types readily, including Fusarium^15^ and gut cells of the fall army worm, *Spodoptera frugiperda*^37^.

BaFA inhibits vacuolar H^+^ ATPase (VATPase), a proton pump responsible for the acidification of endosomal vesicles to facilitate the release of endocytosed contents into the cytoplasm^38^. BaFA has been used to confirm the involvement of clathrin-mediated endocytosis in the uptake of dsRNA in the midgut of the Colorado potato beetle, *L. decemlineata* as well as the hepatopancreas of the whiteleg shrimp, *Litopenaeus vannamei*^34,39^. The BafA-treated hyphal cultures also showed a lack of RNAi, but these cells displayed a different dsRNA distribution than that of the CPZ-treated hyphae. The inhibition of VATPase would prevent acidification of endosomes and release of their contents to the cytoplasm. Hence, in the BafA-treated hyphae, we observed intracellular fluorescence of the fluorescein-labeled dsRNA in a punctate pattern, as the dsRNA remained within endosomal vesicles, and failed to induce RNAi.

In contrast, MBCD did not alter the distribution of fluorescein-labeled dsRNA within the cells, relative to the negative control, and nor did it have a negative effect on RNAi of our reference genes. MBCD is known to affect caveolae-dependent endocytosis^40^, and as we observed no significant reduction of RNAi in the MBCD-treated hyphae, caveolae-dependent endocytosis appears to play no major role in dsRNA uptake. While we cannot rule out that other clathrin-independent processes, such as RhoA-regulated or CDC42-regulated endocytosis^41^ may have minor roles in dsRNA uptake, they have not previously been implicated in dsRNA uptake in other organisms. Given the strong response observed using CME inhibitors, CME is clearly a dominant player in the uptake process.

To validate CME’s involvement in dsRNA uptake in Sclerotinia, we also used an RNAi-of-RNAi approach to identify key players in this endocytosis pathway. By knocking down transcripts of genes encoding CME pathway proteins, we significantly reduced or completely abolished the cells’ abilities to subsequently initiate RNAi of the reference gene. Although we did not survey all possible CME-associated genes, we identified several components of this endocytosis pathway, from early vesicle formation to late endosomes, that are required for dsRNA uptake. The receptor(s) that bind the dsRNA have yet to be identified, but we confirmed that clathrin (or more specifically, the heavy chain of the clathrin complex, CHC) and a component of the clathrin adaptor protein 2 (AP2) complex are essential for dsRNA uptake. Similarly, FCHO1, a protein with membrane binding and bending activity, and amphiphysin (Amph), a protein that recruits dynamin to facilitate the formation and budding process of the endocytic vesicle, respectively, were also observed to be essential for dsRNA uptake, as their knockdown abolished RNAi of the reference genes. Proteins associated with assembly and functioning of late endosomes, including an ADP ribosylation factor-like 1 protein (Arf72A) and a Vacuolar H + ATPase, were also all confirmed as essential for effective RNAi, which suggests that the release of the dsRNA to the cytoplasm occurs late in the endocytic pathway.

The live cell Sclerotinia imaging in the presence of fluorescently-labeled dsRNA suggests that uptake does not occur evenly along the fungal hyphae, but instead occurs predominantly at the hyphal tip of actively growing branches of hyphae. Similarly, the localized reduction of eGFP fluorescence in our transgenic strain of Sclerotinia near the tips of the growing hyphae is supporting evidence that this region is a site of dsRNA entry, facilitating localized gene knockdown effects, followed by a gradual reduction of eGFP fluorescence over the length of hyphae, as the silencing signals spread. The fungal hyphal tip is the site of polarized cell growth, supported by tip-directed transport of secretary vesicles containing a variety of lytic enzymes used to facilitate invasion into a host plant’s tissues^42^. The tip of the hyphae is seen as a site of relatively rapid membrane deposition as vesicles continually fuse with the plasma membrane to exocytose their contents^43^. An endocytic collar, located directly behind the hyphal tip functions to recycle the excess membrane produced by exocytosis. Since the chitinous cell wall must continually expand as the hypha grows, it is thinner at the tip, enabling extracellular materials to easily reach the endocytic collar, which is considered to be the site where the majority of endocytosis occurs in filamentous fungi^44^. Once entering the cell, dsRNA can be transported short distances between fungal cells through septal pores^45^. It is also worth noting that Sclerotinia contains sequences highly similar to RNA-dependent RNA polymerases (RdRps) found in *Verticillium nonalfalfae.* RdRps have been implicated in generating secondary small interfering RNAs (siRNAs) by using the primary siRNAs of an RNAi response as primers to generate more dsRNA using the target mRNA as the template^46^. It will be of interest to determine whether Sclerotinia’s RdRp can contribute to amplification of the RNAi signal, which could help reduce the amount of dsRNA needed to control this fungus effectively.

We observed significant, but not complete knockdown of the targeted genes with the doses applied in this study. If CME is insufficient to deliver effective doses of dsRNA to all cells, and certainly the live cell imaging suggests that the uptake process is not uniform across all hyphae, alternative dsRNA delivery methods may help complement or even circumvent the CME process. In other systems, combining dsRNA with carrier molecules has been shown to enhance uptake. Lipid-based delivery methods have been investigated extensively in human therapeutics, but have also been tested in the fungus *Aspergillus flavus*^47,48^. Increasing the stability of the molecule is also important. In plants, dsRNA bound to clay nanosheets remained intact for up to 30 days in planta in addition to increasing uptake^49^. An approach such as this could be applicable for Sclerotinia and other antifungal dsRNA applications.

In this study, and in one of our previous studies^18^, we demonstrated effective RNAi-mediated knockdown of transcripts in Sclerotinia through exogenous dsRNA applications*.* Other research groups have similarly observed RNAi in other fungal species such as *F. graminearum*^15^ and *B. cinerea*^16^, where transcript reductions resulted in reductions in pathogenicity following dsRNA treatments. In these two other species, along with *S. sclerotiorum* studied here, the dsRNA can obviously enter the fungal cells, but in some fungi, dsRNA does not appear to readily enter the cells. For example, *Zymoseptoria tritici* lacks the ability to take up externally applied fluorescent dsRNAs in vitro^50^. A BLAST search of its genome suggests that this fungus has the key genes necessary for clathrin-mediated endocytosis and RNAi, but it may lack the necessary, but still unidentified, dsRNA receptors needed to trigger CME-mediated uptake of dsRNA. In contrast, *Ustilago maydis* appears to have lost the genes encoding key components of the canonical RNAi pathway such as DCL, AGOs and RdRps^51^. These species, however, may be the exception, as the majority of fungi examined to date contain functional dsRNA uptake and RNAi pathways. Indeed, a steadily growing number of species have been shown to be susceptible to either SIGS or host-induced gene silencing (HIGS)-based control methods^7^.

The ability to deliver dsRNA to various pathogenic fungi has driven many research groups to consider the development of novel dsRNA-based fungicides. The prospect of developing fungicides with high specificity for target species, without adversely affecting non-target species, is a strong motivator to develop this technology. However, with every new pesticide developed, there is the inevitable development of resistance to the pesticide that will arise over time. Due to its sequence-specificity, overcoming small nucleotide mutations is relatively easy to overcome with dsRNA-based pesticides, as there are potentially many different genes that could be targeted to control the pathogenic fungus^18^. However, of greater concern is the possibility that the fungus could develop resistance to many different dsRNAs. Zotti and Smagghe^52^ suggested that one such problematic mode of resistance is alteration in dsRNA uptake mechanisms. This type of resistance has already been identified in a laboratory-selected population of corn rootworms (*D. virgifera*). Within 11 generations of selection on progressively higher doses of dsRNA, Khajuria et al.^20^ produced insects with > 130-fold resistance levels. The resistance was attributed to reduced gut luminal uptake, although the nature of the resistance mutations has yet to be identified. Clearly, resistance due to impaired dsRNA uptake can occur, and if dsRNAs are to be used as biocides, understanding uptake mechanisms will aid in avoiding rapid development of resistance in the field.

Taken together, our results indicate that clathrin-dependent endocytosis is likely responsible for the uptake of dsRNAs in *S. sclerotiorum*. CME has been known to be an integral player in the pathway for the internalization of extra membrane, lipids and receptor-bound macromolecules within eukaryotic organisms and is critical for numerous biological processes such as nutrient acquisition and cell signalling^53^. In Fig. 3a, we adapted the general CME type mechanism to display how we believe dsRNA is internalized within Sclerotinia. The process includes dsRNA molecules first binding to unknown receptor proteins on the cell surface. It has been shown that in some insect species, including S2 cells of Drosophila, scavenger-like receptors are responsible for the binding of dsRNA^30,54^. In humans, Toll-like receptor 3, in addition to class-A type scavenger receptors, specifically SR-AI/II and SCARA 3/4/5 are all able to bind dsRNA^55,56^. However, to our knowledge, no homologues of these receptors exist within Sclerotinia. Once bound, the dsRNA and its receptor interact with clathrin and its adaptor protein complex (AP2) to form a coated pit, which invaginates to form a vesicle. The clathrin sheath uncoats and the vesicle fuses with an early endosome, which finally matures into a late endosome. In this schematic, the dsRNA molecules must release from the endosomes before they mature into functional lysosomes and enter into the cytoplasm to trigger RNAi machinery to perform dsRNA processing and targeted transcript knockdown. While we have shown that CME is the predominant mode of uptake, there are several questions that remain unanswered as we continue to develop species-specific molecular fungicides. We now know how the dsRNA enters the cell but know little about the fate of the molecule once inside, how it is dispersed through the fungus, and whether barriers exist that limit its movements. Identifying other components of the uptake machinery, including the dsRNA receptors, and a closer examination of what dsRNA uptake or dispersal machinery is missing in RNAi-refractory fungal species is a good first step to understanding how resistance to dsRNA might arise, and could help prolong the utility of the technology in phytopathogen control programs.

## Conclusion

RNAi technologies offer the promise to provide a new generation of species-specific, environmentally safe fungicides. To maximize this potential, we need to understand how dsRNAs can be delivered to the pathogenic fungi in sufficient quantities to induce strong knockdown of the target genes. In this study, we demonstrated that CME is an effective dsRNA uptake mechanism in the phytopathogen Sclerotinia. Understanding the cellular machinery involved in dsRNA uptake in fungi will help in the design of more efficient dsRNA delivery formulations and in the development of effective strategies to overcome the possible development of fungal strains that are resistant to dsRNA uptake.

## Materials and methods

### Sclerotinia culture

*Sclerotinia sclerotiorum* cultures were obtained from the Morden Research and Development Centre, Agriculture and Agri-Food Canada, Morden, MB, Canada. Ascospore-bearing apothecia were generated from sclerotia germinated carpogenically on specialized germination medium (54 g cornmeal, 3.5 g vermiculite, 37.5 mL of 1% casamino acids and 1% yeast extract) and incubated on wet sand at 20 °C^57^. After isolation, ascospores were stored in aluminum foil packets at 4 °C. Ascospores (1 × 10^5^ mL^−1^) were plated on potato dextrose agar (PDA) to germinate. After 2 days’ growth, mycelia were harvested from the plates using a P1000 pipette tip to collect agar plugs containing actively growing fungus for use in in vitro liquid culture growth experiments, as described below.

### Design and in vitro synthesis of dsRNAs

In vitro synthesis of Sclerotinia dsRNAs were designed according to the methods of McLoughlin et al.^18^. Reference genes targeted to monitor RNAi-mediated knockdown in this study were selected from a subset of genes from McLoughlin et al.^18^ that showed only minimal dsRNA-mediated death after 3 days’ exposure to dsRNA, but displayed moderate (> 60%) transcript knockdown. Genes encoding proteins associated with endocytosis were identified from the *S. sclerotiorum* transcriptome based on > 60% amino acid conservation to other annotated fungal species such as *Aspergillus clavatus*, using BLASTP (https://blast.ncbi.nlm.nih.gov/Blast.cgi?PAGE=Proteins). Gene function and percent identity to an annotated fungal species can be found in Supplementary Table 1. Primers (Supplementary Table 2) were designed using Primer BLAST (https://www.ncbi.nlm.nih.gov/tools/primer-blast/) to create PCR gene fragments between 200 and 500 bp in length. Target sequences were amplified using Phusion Taq (Thermo Scientific, Waltham, MA, USA) from Sclerotinia cDNA synthesized using the RNeasy Plant Extraction kit (Qiagen, Germantown, MD, USA), RNase Free DNase set (Qiagen, Germantown, MD, USA) and qScript cDNA synthesis kit (Quantabio, Beverly, MA, USA). PCR products were digested using FastDigest KpnI and XhoI (Thermo Scientific, Waltham, MA, USA) and ligated into the similarly digested pL4440 vector using T4 Ligase (Invitrogen, Carlsbad, CA, USA). Primers (F: CAACCTGGCTTATCGAA; R: TAAAACGACGGCCAGTGA) were designed to amplify the T7 promotors flanking the insert and dsRNA was synthesized via T7 reverse transcription using the MEGAscript RNAi kit (Thermo Scientific, Waltham, MA, USA).

### In vitro dsRNA fluorescence labelling for live cell imaging and confocal microscopy

Ss-ThioR dsRNA was labeled with 12-UTP fluorescein and synthesized using the Fluorescein RNA Labeling Mix (Sigma-Aldrich, Oakville, ON, CA). For whole organism labelling, live cell imaging using the ImageXpress plate system (Molecular Devices, San Jose, CA, USA) was used. Ascospores [10 μL of 10^6^ ascospores/mL solution in potato dextrose broth (PDB)] were applied to the center of each well of 12-well culture plates containing 400 μL of ¼ × PDA. Imaging of the fungal growth began 14 h post germination. Fluorescein-labelled Ss-ThioR dsRNA (200 ng) was pipetted directly onto the site of ascospore inoculation immediately prior to imaging. Images were captured in a four by four grid in each well at an excitation/emission of 488 nm and 525 nm wavelengths every 20 min for 24 h to examine fluorescein-labelled dsRNA uptake. A Zeiss LSM 700 confocal microscope (Jena, DEU) was used to study dsRNA distribution within Sclerotinia hyphae. One mm agar plugs from the edge of 3-day old PDA cultures were incubated for 4 h to examine the earlier stages of uptake and 8 h for the later stages in PDB plus F-Ss-ThioR (200 ng). To correct for possible artificial emitted fluorescence, laser gain settings were adjusted to 650 and 350 for fluorescein and T-PMT respectively, in the Zen Black software using untreated controls as the baseline. The same settings were applied to all samples. All microscopic analyses were repeated at least three times, with similar results.

### DsRNA uptake inhibition using chemical inhibition and RNAi of RNAi experiments

To examine the role of endocytosis in dsRNA uptake, in vitro liquid cultures of *Sclerotinia* were used. Three 1 mm agar plugs of Sclerotinia were incubated in 3 mL potato dextrose broth plus 100 μg mL^−1^ ampicillin (Sigma-Aldrich, Oakville, ON, CA) in 15 mL culture tubes. Next, 20 μM chlorpromazine (dissolved in ddH_2_O), 0.2 μM bafilomycin A1 (dissolved in DMSO) or 2 mM methyl-beta-cyclodextrin (dissolved in ddH_2_O) was added to each tube. Two hours after inhibitor inoculation, cultures were treated with 500 ng mL^−1^ Ss-ThioR or Ss-TIM44 dsRNAs and allowed to incubate for 48 h at 22 °C on a shaking incubator, wrapped in aluminum foil. Negative controls were treated with a corresponding volume of water in place of the dsRNA treatment. We and others have shown previously that there is no statistical difference when using controls either treated with water or a non-target dsRNA molecule such as GFP^18,58^. Cultures were then thoroughly rinsed with ddH_2_O to remove liquid media, and the solid hyphal mass was flash frozen in liquid nitrogen and RNA extracted and cDNA synthesized as described above. Transcript levels of these two genes were determined using qRT-PCR on the Bio-Rad CFX96 Connect Real-Time system using PerfeCTa SYBR (Quantabio, Beverly, MA, USA) according to the manufacturer’s protocol. Relative mRNA abundance was calculated using the ΔΔCq method using Sac7 (SS1G_12350) as the housekeeping control. As the efficiencies of all the primers used in this study ranged between 90 and 110% (Supplementary Table 3), a single control gene was considered sufficient. Melt-curve analyses confirmed that only a single product was produced in all RT-PCR reactions.

To identify specific genes involved in the endocytosis process, an RNAi-of-RNAi approach was taken, whereby Sclerotinia was first treated with dsRNA targeting genes associated with endocytosis, and later treated with Ss-ThioR dsRNA. Liquid in vitro cultures of Sclerotinia, as described above, were treated with 500 ng mL^−1^ of each endocytosis target dsRNA, and after four days growth in a shaking incubator at 22 °C, 500 ng mL^−1^ Ss-ThioR dsRNA was added. After an additional 48 h, RNA was extracted and both the endocytic gene and the reference genes’ transcript accumulations were measured as described above.

### Sclerotinia transformation

Transformation was performed with the pCT74 plasmid (Nova Lifetech Ltd, HK) using polyelthylene glycol (PEG)-mediated transformation of protoplasts according to the methods of de Silva et al.^59^. Briefly, approximately 1 g of hyphae was harvested from in vitro cultures and incubated with 200 mg driselase (Sigma-Aldrich, Oakville, ON, CA) that had been resuspended in 0.7 M NaCl. After 3.5 h incubation at 24 °C in driselase to produce protoplasts, the mixture was filtered through sterile cheesecloth and the protoplasts were collected by centrifugation at 400 × *g* and resuspended in STC (20% sucrose, 10 mM Tris–HCl pH 8.0, and 50 mM CaCl_2_). A total of 10 μg of plasmid DNA was added to the protoplasts and they were incubated on ice for 30 min. PEG-4000 (60% w/v) in STC buffer was added to the protoplast mixture and incubated on ice for an additional 5 min. Protoplasts were then re-isolated via centrifugation and plated onto PDA plates. After 2 h, the plates were overlaid with molten PDA plus 100 μg/mL hygromycin B (Thermo Scientific, Waltham, MA, USA). As the pCT74 plasmid contains a GFP reporter gene, putative transformants were examined for their ability to fluoresce at excitation/emission wavelengths of 488 nm and 510 nm, respectively, and successful transformants were re-cultured on fresh hygromycin-augmented PDA several times to ensure purity of the culture. One mm plugs from successful transformants were grown in PBD plus 100 μg/mL hygromycin B inoculated with 1,000 ng mL^−1^ of Ss-eGFP dsRNA with or without the addition of 20 μM chlorpromazine for four days. Mycelia were imaged using the Zeiss Axioscope A1 (Jena, DEU) and the extent of GFP transcript knockdown was quantified using qRT-PCR as described above using primers designed around the eGFP mRNA and using Sac7 as the housekeeping control.

### Data analysis

To analyze the data, JASP (jasp-stats.org) software was used to compute hypothesis testing^60^. To determine whether dsRNA-induced gene knock down significantly differed from the control, student’s *t*-tests were performed with a Bonferroni correction to the level of significance of each comparison.

## Supplementary information

Supplementary Figure S1.

Supplementary Legends.

Supplementary Table 1.

Supplementary Table 2.

Supplementary Table 3.
