# Supplementary material for: Clathrin mediated endocytosis is involved in the uptake of exogenous double-stranded RNA in the white mold phytopathogen Sclerotinia sclerotiorum
Source: Sci Rep. 2020 Jul 29;10:12773. doi: 10.1038/s41598-020-69771-9 (PMC7391711; doi:10.1038/s41598-020-69771-9)

Clathrin Mediated Endocytosis is Involved in the Uptake of Exogenous double-stranded RNA in the White Mold Phytopathogen *Sclerotinia sclerotiorum*

Nick Wytinck[1], Daniel S Sullivan[1], Kirsten T Biggar[1], Leandro Crisostomo[2], Peter Pelka[2], Mark F Belmonte[1] and Steve Whyard[1,*]
[1]University of Manitoba, Department of Biological Sciences, Winnipeg, R3T 2N2, Canada
[2]University of Manitoba, Department of Microbiology, Winnipeg, R3T 2N2, Canada
*Steve.Whyard@umanitoba.ca

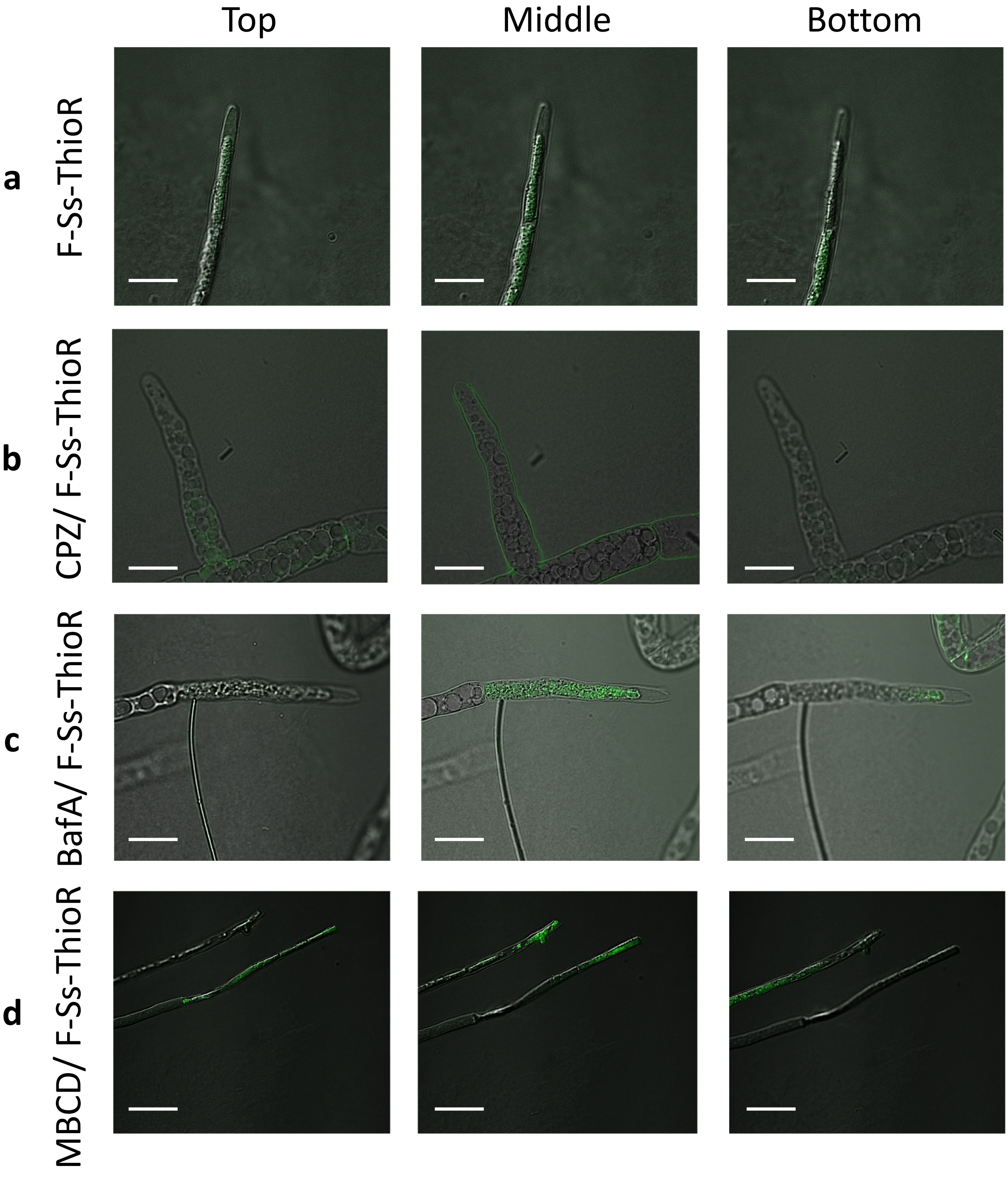

Supplement: Supplementary file 1 — Supplementary Figure S1. [file 41598_2020_69771_MOESM1_ESM.pdf]
