# Supplementary material for: Clathrin mediated endocytosis is involved in the uptake of exogenous double-stranded RNA in the white mold phytopathogen Sclerotinia sclerotiorum
Source: Sci Rep. 2020 Jul 29;10:12773. doi: 10.1038/s41598-020-69771-9 (PMC7391711; doi:10.1038/s41598-020-69771-9)
Supplement: Supplementary file 2 — Supplementary Legends. [file 41598_2020_69771_MOESM2_ESM.docx]

**Figure S1 .** a) *In vitro* cultures were inoculated with 20 $\mu$M chlorpromazine, b) 0.2 $\mu$M Bafilomycin A1, or c) 2 mM methyl-beta-cyclodextrin and two hours later, with 500 ng/mL Ss-ThioR or Ss-TIM44 dsRNA. Confocal microscopy was used to view above and below the focal plane of the specimen to demonstrate that the dsRNA was intracellular and not only bound to the outer surface of the hyphae.

**Supplementary Table 1.** Gene identifications and functions for each of the gene targets used in this study. In order to identify the Sclerotinia copy of each of these genes, amino acid homology to an annotated fungal species, *Aspergillus clavatus*, was used.

**Supplementary Table 2.** Cloning primer sequences as well as dsRNA fragment lengths used in this study.

**Supplementary Table 3.** qRT PCR primer sequences used in this study.
