# Supplementary material for: Clathrin mediated endocytosis is involved in the uptake of exogenous double-stranded RNA in the white mold phytopathogen Sclerotinia sclerotiorum
Source: Sci Rep. 2020 Jul 29;10:12773. doi: 10.1038/s41598-020-69771-9 (PMC7391711; doi:10.1038/s41598-020-69771-9)
Supplement: Supplementary file 3 — Supplementary Table 1. [file 41598_2020_69771_MOESM3_ESM.docx]

| **Ss Gene name** | **Gene Function** | **Ss gene ID** | **Orthologous *Aspergillus* gene ID** | **Amino acid percent identity** |
| --- | --- | --- | --- | --- |
| Ss-ThioR | Thioredoxin reductase | SS1G_05899 | ACLA_051990 | 67% |
| Ss-TIM44 | Mitochondrial inner membrane translocase subunit TIM44 | SS1G_06487 | ACLA_025660 | 77% |
| Ss-CHC | Clathrin heavy chain | SS1G_12840 | ACLA_047060 | 78% |
| Ss-AP2 | Clathrin adaptor protein 2 | SS1G_06066 | ACLA_054080 | 68% |
| Ss-Arf72A | ADP ribosylation factor 72A | SS1G_06730 | ACLA_023910 | 92% |
| Ss-FCHO1 | F-BAR domain protein 1 | SS1G_06645 | ACLA_038110 | 68% |
| Ss-Amph | Amphiphysin | SS1G_00939 | ACLA_041720 | 64% |
| Ss-VATPase | Vacuolar H+ ATPase 16 kDa subunit | SS1G_10240 | ACLA_040240 | 87% |

**Clathrin Mediated Endocytosis is Involved in the Uptake of Exogenous double-stranded RNA in the White Mold Phytopathogen *Sclerotinia sclerotiorum***

Nick Wytinck^1^, Daniel S Sullivan^1^, Kirsten T Biggar^1^, Leandro Crisostomo^2^, Peter Pelka^2^, Mark F Belmonte^1^ and Steve Whyard^1.*^

^1^University of Manitoba, Department of Biological Sciences, Winnipeg, R3T 2N2, Canada

^2^University of Manitoba, Department of Microbiology, Winnipeg, R3T 2N2, Canada

*Steve.Whyard@umanitoba.ca
