# Supplementary material for: Clathrin mediated endocytosis is involved in the uptake of exogenous double-stranded RNA in the white mold phytopathogen Sclerotinia sclerotiorum
Source: Sci Rep. 2020 Jul 29;10:12773. doi: 10.1038/s41598-020-69771-9 (PMC7391711; doi:10.1038/s41598-020-69771-9)
Supplement: Supplementary file 4 — Supplementary Table 2. [file 41598_2020_69771_MOESM4_ESM.docx]

**Clathrin Mediated Endocytosis is Involved in the Uptake of Exogenous double-stranded RNA in the White Mold Phytopathogen *Sclerotinia sclerotiorum***

Nick Wytinck^1^, Daniel S Sullivan^1^, Kirsten T Biggar^1^, Leandro Crisostomo^2^, Peter Pelka^2^, Mark F Belmonte^1^ and Steve Whyard^1.*^

^1^University of Manitoba, Department of Biological Sciences, Winnipeg, R3T 2N2, Canada

^2^University of Manitoba, Department of Microbiology, Winnipeg, R3T 2N2, Canada

*Steve.Whyard@umanitoba.ca

| **Ss gene ID** | **Forward Primer** | **Reverse Primer** | **dsRNA length (bp)** |
| --- | --- | --- | --- |
| Ss-ThioR | GCTCACACTGCTGCCGTATA | CTCCGCAGCACTATCTCCAC | 435 |
| Ss-TIM44 | CGTAGCATATCCGACCGAGT | AGACGGAGAATTGAGCTGCT | 211 |
| Ss-CHC | TTGACCCAAATCAGGCAGCT | ATGGCGACTGGGAAATCGTT | 397 |
| SS-AP2 | GCGATGACCCTGTTCCTTCA | GACCGAGAGAGCAACACCAA | 333 |
| Ss-Arf72A | GGTTGAGGCTCGTGAGGAAT | GCGAGCCATTCCAATCCTTC | 222 |
| Ss-FCHO1 | AGACGAAAGAGCACCAAGGG | TTTCTCAGGTGCTGCGACAT | 261 |
| Ss-Amph | AGCGCCTCAAACGTTCAAAG | TTCTTCACACGCCCTGATCC | 280 |
| Ss-VATPase | CACTTGTTTCGGTGCTGCAT | AGACGTGGTTGTTGAGCTGT | 306 |
| Ss-eGFP | CTGACCTACGGCGTGCAG | GACCATGTGATCGCGCTTCT | 468 |
