# Supplementary material for: Clathrin mediated endocytosis is involved in the uptake of exogenous double-stranded RNA in the white mold phytopathogen Sclerotinia sclerotiorum
Source: Sci Rep. 2020 Jul 29;10:12773. doi: 10.1038/s41598-020-69771-9 (PMC7391711; doi:10.1038/s41598-020-69771-9)
Supplement: Supplementary file 5 — Supplementary Table 3. [file 41598_2020_69771_MOESM5_ESM.docx]

**Clathrin Mediated Endocytosis is Involved in the Uptake of Exogenous double-stranded RNA in the White Mold Phytopathogen *Sclerotinia sclerotiorum***

Nick Wytinck^1^, Daniel S Sullivan^1^, Kirsten T Biggar^1^, Leandro Crisostomo^2^, Peter Pelka^2^, Mark F Belmonte^1^ and Steve Whyard^1.*^

^1^University of Manitoba, Department of Biological Sciences, Winnipeg, R3T 2N2, Canada

^2^University of Manitoba, Department of Microbiology, Winnipeg, R3T 2N2, Canada

*Steve.Whyard@umanitoba.ca

| **Ss gene ID** | **Forward Primer** | **Reverse Primer** | **Efficiency** |
| --- | --- | --- | --- |
| Ss-ThioR | ACACGGTTGGAGGCGAAATT | TCCTCTCCAGTAACGACGTTC | 96% |
| Ss-TIM44 | TACTTGACAAGGCGACGCAA | ACCCACCCTCCGTACCTC | 108% |
| Ss-CHC | GCCTTCCTTAGCGCTGATCT | GCAAGCTCTCATTGTCGCTG | 98% |
| SS-AP2 | AAATGCGGTTTTGTTTGAGG | TTGGATGAACTTCCCGAGTC | 93% |
| Ss-Arf72A | TACAACTATCCCAACCATCGGC | CACCAACATCCCACACGGTA | 95% |
| Ss-FCHO1 | TCGAAGTCAATGCAGCAAAC | CTGTGAATCTGGCCTTGGTT | 92% |
| Ss-Amph | TGATGAACCACCAGATCGAA | CCCTTCTTGAACCAGGGAAT | 98% |
| Ss-VATPase | GTATACGCGCCATTCTTCGG | GCAGCACCGAAACAAGTGAA | 102% |
| Ss-eGFP | AACGAGAAGCGCGATCACAT | TTGTACAGCTCGTCCATGCC | 91% |
